# Ambroxol Hydrochloride Improves Motor Functions and Extends Survival in a Mouse Model of Familial Amyotrophic Lateral Sclerosis

**DOI:** 10.3389/fphar.2019.00883

**Published:** 2019-08-07

**Authors:** Alexandra Bouscary, Cyril Quessada, Althéa Mosbach, Noëlle Callizot, Michael Spedding, Jean-Philippe Loeffler, Alexandre Henriques

**Affiliations:** ^1^Université de Strasbourg, UMR_S 1118, Fédération de Médecine Translationnelle, Strasbourg, France; ^2^INSERM, U1118, Mécanismes Centraux et Périphériques de la Neurodégénérescence, Strasbourg, France; ^3^Neuro-sys SAS, Gardanne, France; ^4^Spedding Research Solutions SAS, Le Vesinet, France

**Keywords:** ambroxol, GBA2, glucocerebrosidase, ALS, neuromuscular junction, glucosylceramide

## Abstract

Amyotrophic lateral sclerosis (ALS) is a multifactorial and fatal neurodegenerative disease. Growing evidence connects sphingolipid metabolism to the pathophysiology of ALS. In particular, levels of ceramides, glucosylceramides, and gangliosides are dysregulated in the central nervous system and at the neuromuscular junctions of both animal models and patients. Glucosylceramide is the main precursor of complex glycosphingolipids that is degraded by lysosomal (GBA1) or non-lysosomal (GBA2) glucocerebrosidase. Here, we report that GBA2, but not GBA1, activity is markedly increased in the spinal cord, of SOD1^G86R^ mice, an animal model of familial ALS, even before disease onset. We therefore investigated the effects of ambroxol hydrochloride, a known GBA2 inhibitor, in SOD1^G86R^ mice. A presymptomatic administration of ambroxol hydrochloride, in the drinking water, delayed disease onset, protecting neuromuscular junctions, and the number of functional spinal motor neurons. When administered at disease onset, ambroxol hydrochloride delayed motor function decline, protected neuromuscular junctions, and extended overall survival of the SOD1^G86R^ mice. In addition, ambroxol hydrochloride improved motor recovery and muscle re-innervation after transient sciatic nerve injury in non-transgenic mice and promoted axonal elongation in an *in vitro* model of motor unit. Our study suggests that ambroxol hydrochloride promotes and protects motor units and improves axonal plasticity, and that this generic compound is a promising drug candidate for ALS.

## Introduction

Amyotrophic lateral sclerosis (ALS) is a fatal neurodegenerative disease characterized by a loss of cortical motor neurons in the motor cortex and spinal motor neurons located in the brainstem and in the spinal cord with denervation. Considered to be the most common motor neuron disease in adults, ALS leads to progressive paralysis, muscle atrophy, fasciculation, and spasticity and affects the central nervous system and peripheral organs (Schmitt et al., 2014). ALS is associated with sporadic forms (90%) and familial form (10%). Mutations on genes encoding superoxide dismutase 1 (SOD1), TAR DNA-binding protein of 43 kDa (TDP-43) and fused in sarcoma (FUS), and repeat expansions in chromosome 9 open reading frame 72 (C9ORF72) (Lattante et al., 2015) are now documented. It is reported that lipid metabolism in ALS patient has a major impact on the disease severity. A high incidence of dyslipidemia and hypermetabolism is present in ALS patients (Desport et al., 2001; Funalot et al., 2009) and hypermetabolism and high low-density lipoprotein (LDL)/high-density lipoprotein (HDL) ratios or high body mass index are associated with better prognosis and slower disease progression (Dupuis et al., 2008; Paganoni et al., 2011; Jésus et al., 2018). The causes of the metabolic dysfunctions in ALS remain unknown and could result from central pathologies combined with peripheral alterations. 

Metabolomic studies have now shown that recent human evolution has marked changes in lipid metabolism in muscle and brain to support increased metabolic activity (Noakes and Spedding, 2012; Bozek et al., 2014). Beyond their role in energy metabolism, lipids and particularly sphingolipids are modulators of cellular signaling pathways and participate in the maintenance and repair of the various components of the motor axis such as neurons and muscles. Our transcriptomic studies on muscle biopsies in ALS patients showed a significant increase in the expression of the UGCG gene (UDP-glucose ceramide glucosyltransferase), encoding the sphingolipid metabolism enzyme that synthesizes glucosylceramide (GlcCer) (Henriques et al., 2015; Dodge, 2017; Henriques et al., 2017). It has been shown in ALS that GlcCer and ceramide levels are deregulated (Cutler et al., 2002; Dodge et al., 2015; Henriques et al., 2015; Henriques et al., 2018). These data suggest that GlcCer plays a key role in the pathophysiology of ALS. We performed a lipidomic analysis of different tissues of SOD1^G86R^ mice, an animal model of ALS, and we observed a complete rearrangement of the main lipid classes, including sphingolipids like GlcCer and ceramides in the muscles and spinal cords of SOD1^G86R^ mice, before the onset of the disease. Inhibition of GlcCer synthesis by administration of a UGCG inhibitor (AMP-DMN) significantly delays functional recovery after sciatic nerve injury (Henriques et al., 2015; Henriques et al., 2018). GlcCer is the precursor of gangliosides, and this hydrolysis is performed by GBA1 and GBA2, two beta-glucocerebrosidases (GCases). 

Our previous results have shown a beneficial effect for SOD1^G86R^ mice after inhibition of GlcCer degradation (Henriques et al., 2017). Partial inhibition of GlcCer degradation with a low dose of conduritol B epoxide (CBE) (10 mg/kg/d) delays disease onset and improves motor functions in presymptomatic and in symptomatic SOD1^G86R^ mice. Pharmacological inhibition of GCase by CBE preserves motor neuron number and the neuromuscular junctions (NMJs) in SOD1^G86R^ mice. Furthermore, CBE promotes recovery after sciatic nerve injury *in vivo* (Henriques et al., 2017). Conversely, a high dose CBE (100 mg/kg/d) induced neuronal toxicity and can be used to inhibit the lysosomal GCase to induce a chemical model of Gaucher’s diseases (Kanfer et al., 1975; Vardi et al., 2016). Indeed, CBE is an inhibitor of lysosomal GCase (GBA1) and, less potently, of the non-lysosomal GCase (GBA2) (Ridley et al., 2013). Moreover, loss-of-function mutations of GBA1 are a major cause of hereditary Parkinson’s Disease (PD), while the activation of beta-GCase increases alpha-synuclein clearance and lysosomal function in dopaminergic neurons (Schapira, 2015; Mazzulli et al., 2016; Stojkovska et al., 2018); thus, there may be a risk of PD with GBA1 inhibitors.

GBA2 is localized at the plasma membrane and as a membrane-associated protein at the Golgi apparatus and at the endoplasmic reticulum (Woeste and Wachten, 2017). Loss of function of GBA2 is associated with hereditary spastic paraplegia suggesting that the regulation of GlcCer at the plasma membrane and/or at intracellular organelles is important for the maintenance of motor functions, even if the role of GBA2 in the central nervous system remains poorly understood. 

Our approach was therefore to inhibit the non-lysosomal GBA2 without inhibiting GBA1. Among the safe molecules able to cross the blood–brain barrier (BBB), ambroxol hydrochloride (AMB) has been extensively studied (Albin and Dauer, 2014; McNeill et al., 2014; Ambrosi et al., 2015; Migdalska‐Richards et al., 2016b; Migdalska‐Richards et al., 2017; O’Regan et al., 2017). In our study, we confirmed that AMB inhibited GBA2 activity. We have then investigated the effects of AMB *in vivo*, in a transgenic model of ALS and on non-transgenic mice to determine whether it could improve the lifespan of SOD1^G86R^ mice and stimulate the plasticity of the neuromuscular junctions (NMJs).

## Results

### GBA2 Activity Is Increased in Pre-Symptomatic SOD1^G86R^ Mice

GlcCer is a precursor of complex glycosphingolipids. It is synthetized from ceramide by the GlcCer synthase and degraded by GBA1 or GBA2, two GCases. We previously reported that inhibition of GCase activity by a low dose of CBE is neuroprotective in the SOD1^G86R^ mice (Henriques et al., 2017). CBE has inhibitory activities on lysosomal and GBA2 activity (Ridley et al., 2013). 

**Figure 1:**
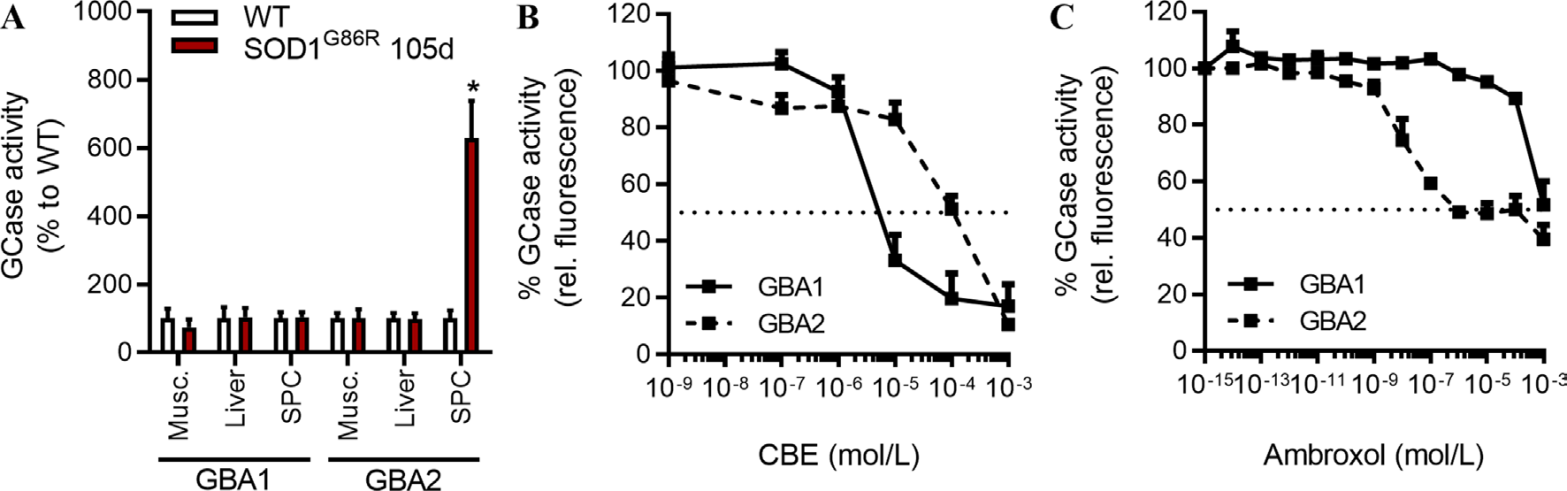
GCase activities in symptomatic SOD1^G86R^ mice. **(A)** GCase activity in different tissues of SOD1^G86R^ and wild type (WT) mice at 105d (*n* = 5–9/tissues, *p < 0.05). **(B)** GCase [glucocerebrosidase (GBA)] activity after conduritol B epoxide (CBE) dose-response (*n* = 6/group). **(C)** GCase (GBA) activity after ambroxol hydrochloride (AMB) dose-response in liver tissue (*n* = 4/group).

Here, we sought to measure GCase activities at specific pH, and in presence or absence of detergent, to determine whether GBA1 (pH = 4.6, with detergent) and GBA2 (pH = 5.8, no detergent) activities are dysregulated in tissues of SOD1^G86R^ mice (Witte et al., 2010). At a symptomatic disease stage, characterized by moderate motor dysfunctions, GBA1 activity was not significantly altered in SOD1^G86R^ when compared to control non-transgenic mice (**Figure 1A**). However, GBA2 activity was strongly increased in the spinal cord of SOD1^G86R^ mice at 105d (symptomatic) but not in muscle or in liver. This increase in GBA2 activity was also observed in presymptomatic SOD1^G86R^ mice (**Supplementary Figure 1A**). The GBA1 activity was not changed at either time point. GBA1 and GBA2 share common enzymatic activity but differ in structure, thus allowing the use of specific inhibitors. The effects of AMB on GCase activities *in vitro* have been compared to those of CBE. As previously reported (Ridley et al., 2013), CBE had a strong inhibitory effect on the GBA1 activity being 10-fold less potent on GBA2 activity (**Figure 1B**). AMB has been reported to inhibit the GBA2 activity (Maegawa et al., 2009), which was confirmed in a dose-dependent manner (**Figure 1C**). Taken together, these results show that GBA2 activity is increased in SOD1^G86R^ mice, already at pre-symptomatic disease stage, and that AMB can inhibit its activity.

### Ambroxol Hydrochloride Delays Disease Onset, Preserve the Integrity of Motor Units, and Increases Survival of SOD1^G86R^ Mice

**Figure 2:**
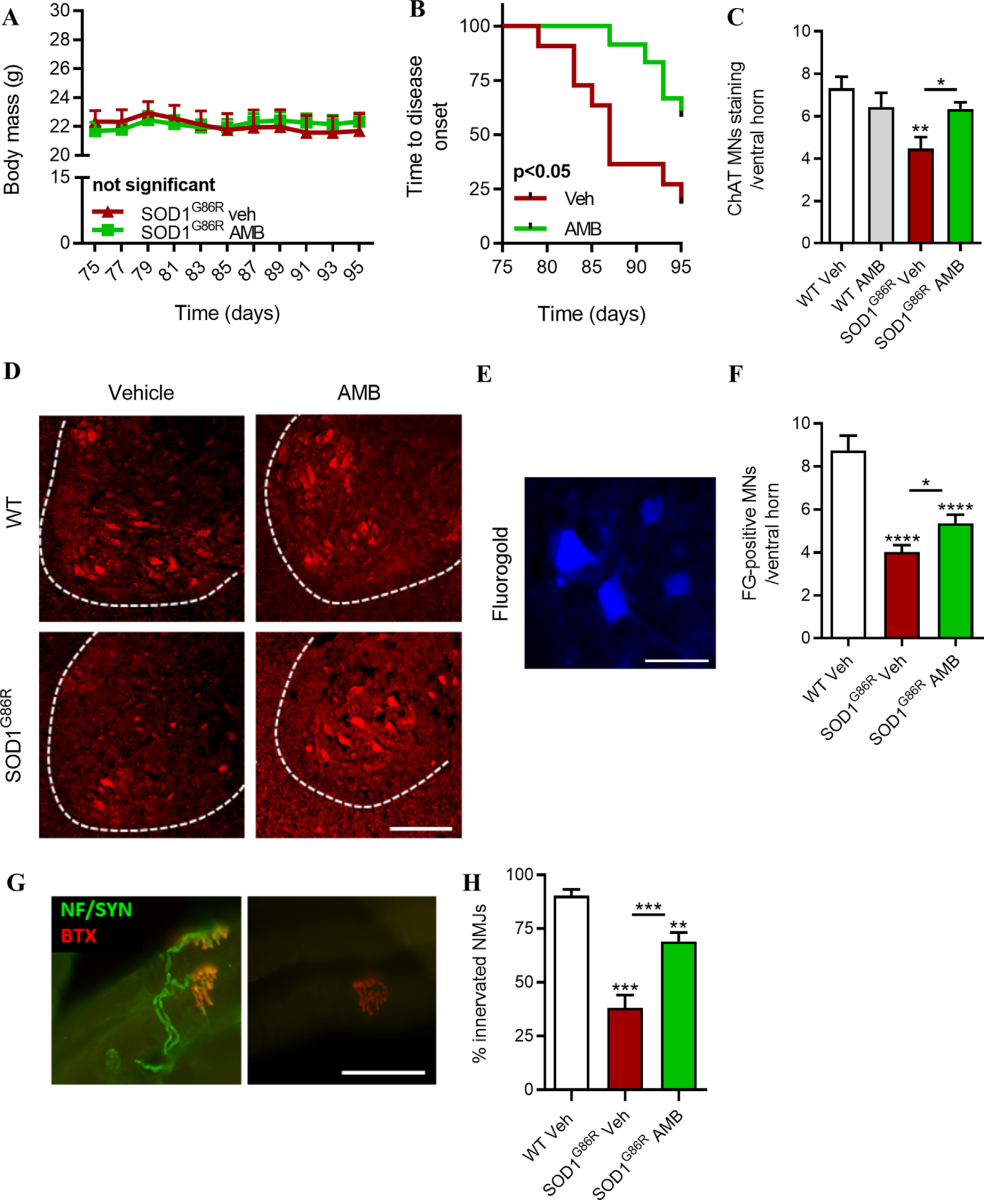
AMB improves motor functions and preserves neuromuscular junction and the functional motor neurons (MNs) in pre-symptomatic SOD1^G86R^ mice. **(A)** Body mass evolution in SOD1^G86R^ mice after AMB treatment (not significant, *n* = 11–12/group). **(B)** Kaplan–Meier showing time to onset of muscle strength loss in SOD1^G86R^ mice (*p* < 0.05, *n* = 11–12/group). **(C)** Quantification of choline acetyl transferase (ChAT)-positive cells located in the ventral horn of the spinal cord and having a size bigger than 400 μm^2^ (*n* = 5/group). **(D)** Representative pictures of the ventral horn of the L1–L3 lumbar area, after immunostaining with ChAT (red), a marker specific for spinal motor neurons. Ventral horns are delimited by dashed lines. Scale bar = 100 μm. **(E)** Representative picture of retrogradely labeled spinal MNs after fluorogold (FG) injection in hindlimb muscles. Scale bar = 50 µm. **(F)** Quantification of FG-positive MNs in ventral horn of the spinal cord (*n* = 10/group, *p < 0.05; ****p < 0.00001, one-way ANOVA test). **(G)** Representative pictures of innervated (left panel) and denervated (right panel) of neuromuscular junctions in SOD1^G86R^ mice (bungarotoxin, BTX, red; neurofilament and synaptophysin, green). Scale bar = 50 μm. **(H)** Neuromuscular junctions (NMJ) integrity in tibialis anterior muscle (**p < 0.01; ***p < 0.001).

AMB was administrated to presymptomatic SOD1^G86R^ mice, to determine whether it could influence disease onset and loss of motor functions in a preventive manner. Grip strength was used as an indicator of muscle strength. SOD1^G86R^ mice were treated from 75 days to 95 days of age, and body mass and muscle strength were evaluated every other day (**Figure 2**). In the first cohort of presymptomatic SOD1^G86R^ mice, AMB had no significant effects on the body mass of treated mice (**Figure 2A**); however, it strongly improved the muscle strength of SOD1^G86R^ mice and significantly delayed of disease onset, defined as a drop of more than 20% of the mouse maximal strength (**Figure 2B**). These results were replicated in a separate cohort of SOD1^G86R^ mice, using the same experimental conditions (**Supplementary Figure 2**). Indeed, an improved muscle strength and a delayed disease onset were observed after AMB administration in the second cohort.

At 95 days of age, neurodegeneration of motor neurons is detected in the lumbar region of the spinal cord of SOD1^G86R^ mice (Henriques et al., 2017). As compared to the WT mice, 40% of motor neurons (MNs) are lost at this time point. The number of MNs in the lumbar region of the spinal cord was significantly higher in SOD1^G86R^ mice treated with AMB (**Figures 2C, D**), suggesting that presymptomatic administration of AMB delays neurodegeneration. In order to determine whether the spared MNs have axonal projection to hind limb muscles, fluorogold, a retrograde tracer, has been injected in gastrocnemius and tibialis anterior muscles. SOD1^G86R^ mice treated with AMB had more MNs-positive for fluorogold (**Figures 2E, F**).

Muscle strength is dependent on the innervation status of muscles. The integrity of NMJs was assessed by immunohistochemistry with the overlapping of post-synaptic cluster of nicotinic acetylcholine receptors (nAChR) and the axonal markers neurofilament and synaptophysin. AMB has significantly protected muscle innervation in SOD1^G86R^ mice, as they had almost two times more NMJs than the vehicle group, at 95 days of age (**Figure 2G, H**). These results suggest that presymptomatic administration of AMB delays disease onset by improving the integrity of the motor units.

**Figure 3:**
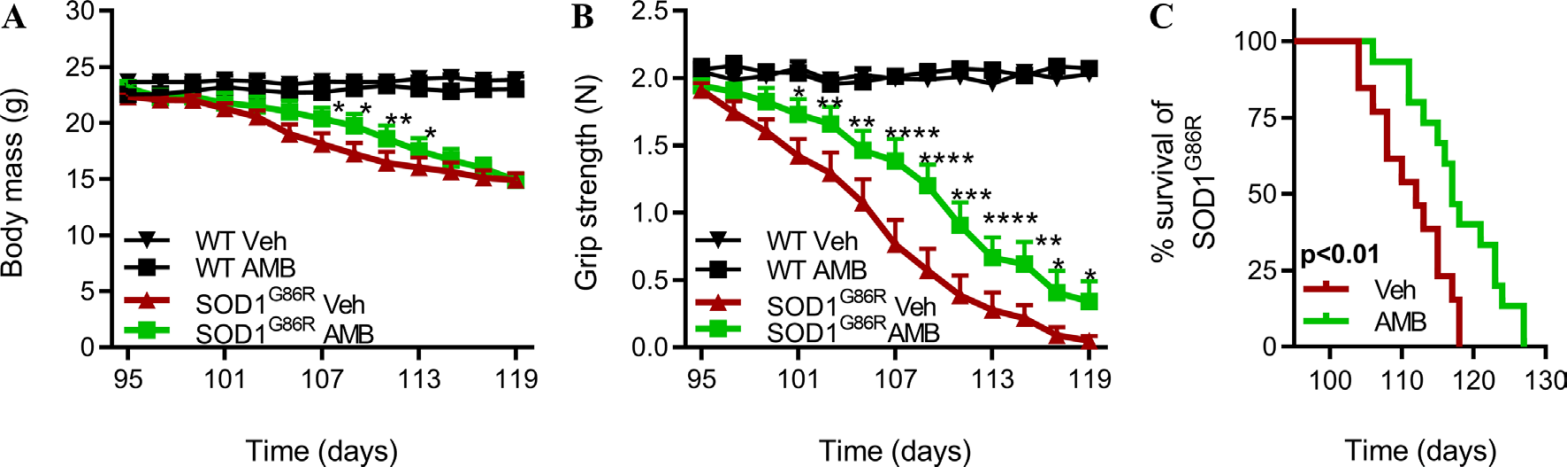
AMB delays disease onset and improves motor functions in symptomatic SOD1^G86R^ mice. **(A)** Body mass evolution in SOD1^G86R^ and WT mice (*n* = 12–15/group). **(B)** Muscle strength evolution in SOD1^G86R^ and WT (*n* = 12–15/group). **(C)** Kaplan–Meier analysis of SOD1^G86R^ mice survival (*n* = 13–15/group, *p < 0.05; **p < 0.01; ***p < 0.001; ****p < 0.0001).

Next, we sought to determine whether a later administration of AMB, at disease onset, could slow down disease progression and improve survival of SOD1^G86R^ mice. AMB was administrated to SOD1^G86R^ mice and wild-type littermates, starting at 95 days of age. From 95 days of age, motor symptoms progress rapidly in SOD1^G86R^ mice, and they reach disease end stage around 110 days of age. Body mass, muscle strength, and overall survival were monitored to follow the progression of ALS symptoms (**Figure 3**). After administration of AMB, the decline in body mass was limited, and SOD1^G86R^ mice were heavier, although this effect was transient (**Figure 3A**). A clear improvement of muscle strength was observed, starting from day 101, in SOD1^G86R^ mice treated with AMB when compared to the SOD1^G86R^ vehicle group (**Figure 3B**). Most importantly, survival of SOD1^G86R^ mice was significantly increased by 6 days after administration of AMB (**Figure 3C**).

Altogether, these results show that AMB delays disease progression when the treatment is initiated at disease onset. 

### Ambroxol Hydrochloride Improves Motor Recovery After Sciatic Nerve Injury in Non-Transgenic Mice

We have previously demonstrated that partial inhibition of GCase activity with CBE improves axonal elongation *in vitro* and *in vivo* recoveries after sciatic nerve injury (Henriques et al., 2017).

**Figure 4:**
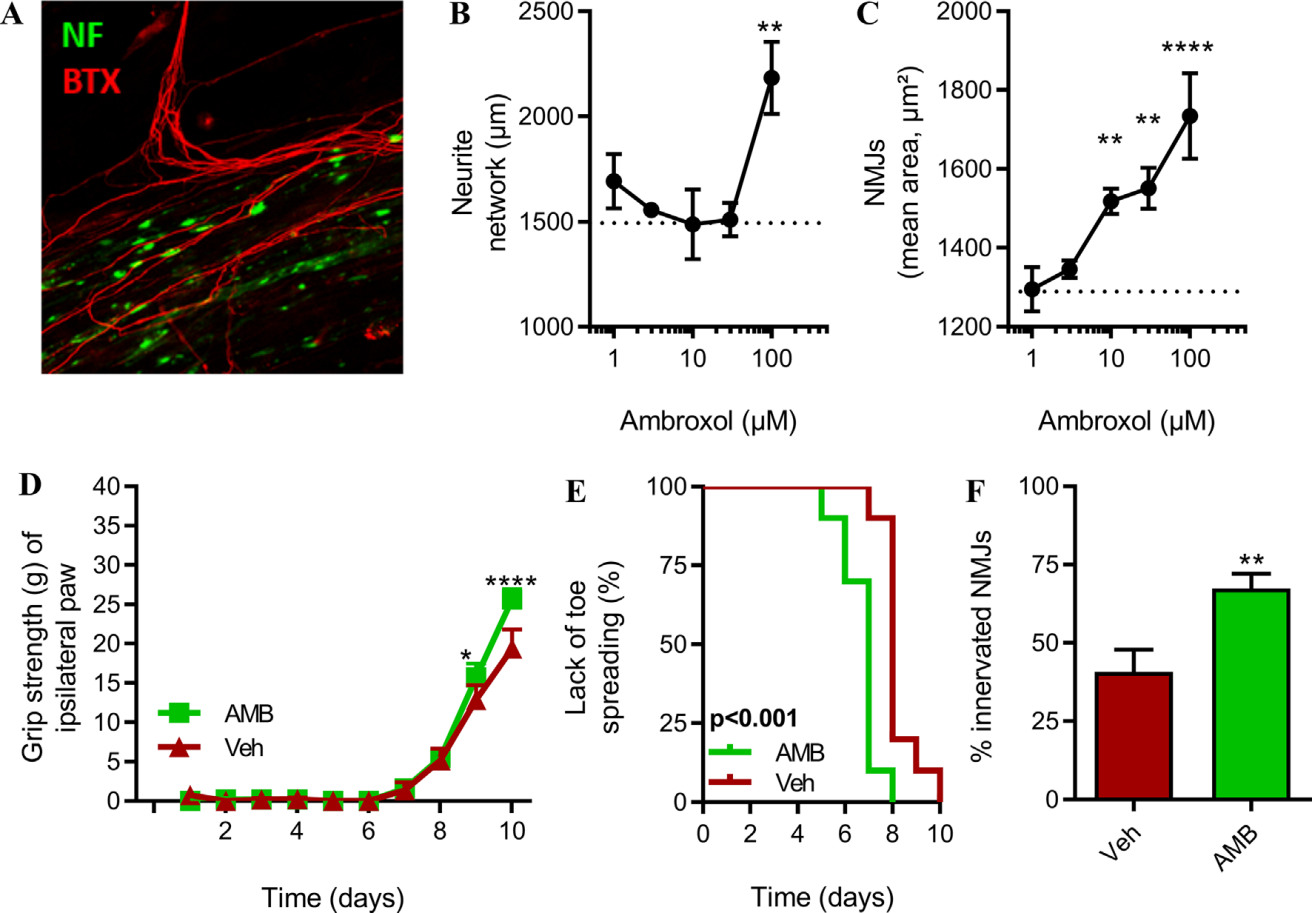
Inhibition of GlcCer degradation by AMB improves *in vivo* axonal plasticity and nerve regeneration after sciatic nerve crush study in non-transgenic mice. **(A)** Representative picture of *in vitro* neuromuscular junctions (NMJs). **(B)** Total length of neurofilament-positive neurites and **(C)** total area NMJs (*n* = 5/group). **(D)** Muscle strength of ipsilateral hind paws (*n* = 10/group, *p < 0.05; ****p < 0.0001). **(E)** Kaplan–Meier showing time to observable toe spreading after sciatic nerve injury (*n* = 10/group, *p* < 0.001). **(F)** NMJ integrity in tibialis anterior muscle (*n* = 10/group, **p < 0.01).

We hypothesized that AMB could exert similar pro-regenerative effects. First, we investigated whether AMB could enhance axonal elongation and the formation of NMJs, in an *in vitro* model of motor units, based on a co-culture of myoblasts and spinal cord explants (Combes et al., 2015). This *in vitro* model allows the maturation of axons and the formation of NMJs, as observed by muscle contraction triggered by the application of acetylcholine. The presence of NMJs was confirmed by the colocalization of the axonal neurofilament and the presence of post-synaptic nicotinic acetylcholine receptors (**Figure 4A**, **Supplementary Figure 3**).

The neurite network was significantly longer in presence of the highest dose of AMB (100 µM) (**Figure 4B**). This effect corresponds to a +45% increase when compared to the vehicle group. Lower doses of AMB did not promote neurite elongation. 

AMB was able to significantly increase the number of *in vitro* NMJs, determined by the number clusterized nAChRs, in a linear dose-dependent manner, at doses ranging from 10 to 100 µM. The effect of AMB was therefore stronger on the formation of NMJs than on the elongation of axons, in this model (**Figure 4C**).

To further evaluate the pro-regenerative effect of AMB on the motor units, we have subjected non-transgenic mice to peripheral nerve injury, to follow-up functional recovery, such as muscle strength and toe spreading, and the number of functional NMJs after treatment with AMB. Upon treatment, mice recovered significantly faster when compared to the control group. Indeed, on days 9 and 10, muscle strength of the injured hindlimb was statistically higher after application of AMB (**Figure 4D**). In a second and independent cohort, we have confirmed the beneficial effect of AMB (3 mM) on functional recovery after sciatic injury. The regenerative effect of AMB was also observed at a dose of 1 mM in this model (**Supplementary Figure 4**). Spontaneous toe spreading is lost after hindlimb muscle denervation. The recovery of toe spreading is a proxy for early sign of reinnervation and was monitored every day. AMB caused a modest but significant effect, as the mice recovered between 1 to 2 days earlier as compared to the control group (**Figure 4E**). Most importantly, 10 days after injury, the number of innervated neuromuscular junctions was 70% greater after application of AMB, suggesting that AMB promotes the formation of new NMJs, which may strongly participate in the pro-regenerative effect of this compound (**Figure 4F**).

## Discussion

### Glycosphingolipids and Motor Axis

Dysregulation of GlcCer and other glycosphingolipids (e.g., GM1a) was previously reported by us and others in ALS patients and in animal models (Dodge et al., 2015; Henriques et al., 2015; Dodge, 2017; Henriques et al., 2017; Henriques et al., 2018).

GlcCer is degraded by GBA1 or GBA2 beta-GCases. Growing evidence suggests a tight connection between GBA2 activity and motor functions, as full loss of function of GBA2 is a cause of hereditary spastic paraplegia type 46 (SPG46) (Woeste and Wachten, 2017; Woeste et al., 2019). Conversely, pharmacological inhibition of GBA2 activity has been shown to improve the motor phenotype (e.g., motor coordination) of an animal model of Niemann’s Pick disease (Marques et al., 2015). Moreover, GM1a, a downstream metabolite of GlcCer, is detected on the cell surface of motor axons and at nerve endings in NMJs. The presence of anti-GM1 autoantibodies has been associated with multifocal motor neuropathy and the acute motor axonal form of Guillain-Barré, suggesting a key role for glycosphingolipids in the maintenance of motor axons and possibly NMJs. 

We have shown that inhibition of GCase by CBE causes a concentration-dependent increase in GlcCer, and in the downstream glycosphingolipid GM1a, which was associated with pro-survival and pro-regenerative effects. Treatment with CBE fully prevented loss of GM1a/CTB staining at NMJs in tibialis anterior muscle of SOD1^G86R^ mice, associated with delayed loss in grip strength (Henriques et al., 2017). Interestingly, the inhibition of GlcCer synthase (GCS) accelerates functional decline. Inhibition of GCS also delays the recovery to spinal crush in non-transgenic mice. Thus, a coherent picture emerges in ALS where inhibiting GCase activity and increasing the pool of glycosphingolipids, presumably at the NMJs, are beneficial.

### Ambroxol Improved Motor Function in SOD1^G86R^ Mice

Our results showed that GBA2 activity is increased in the spinal cord of SOD1^G86R^ mice, even at the presymptomatic disease stage, which could impair the metabolism of glycosphingolipids and later contribute to the development of motor dysfunctions. Here, we propose ambroxol as a drug candidate for ALS. Ambroxol is known to inhibit of GBA2 activity, mainly catalyzed by GBA2. By inhibiting GBA2, we aimed to prevent the hydrolysis of GlcCer located outside the lysosome, mainly at the plasma membrane, the endoplasmic reticulum, and/or at the Golgi apparatus (Maegawa et al., 2009; Shanmuganathan and Britz-McKibbin, 2011). Our study showed that ambroxol delayed the decline of motor functions and protected motor neurons from degeneration in a transgenic animal model of ALS. Most importantly, ambroxol was able to maintain the functionality of spinal motor neurons in the SOD1^G86R^ mice. For these promising pre-clinical effects, ambroxol has recently been given orphan drug status for ALS by the European Medical Agency.

### Clinical Relevance of Ambroxol for ALS

Ambroxol is marketed as an expectorant and mucolytic for lung diseases and for sore throat, with local anesthetic effects. In the CNS, ambroxol is potentially an inhibitor of sodium (Nav1.8) and calcium channels, with claimed anti-glutamatergic properties. Evidence suggests that ambroxol has direct anti-oxidative properties which could translate into beneficial effects in ALS (reviewed by Weiser, 2008). 

In addition to its effects on GBA2, ambroxol is also known to bind the enzyme GBA1 in the cytosol and to correct enzyme folding and to improve its addressing to the acidic environment of the lysosome where the drug dissociates, thereby increasing GBA1 activity. The chaperone effect on ambroxol on GBA1 is combined with a positive modulation of GBA1 expression level (Magalhaes et al., 2018). It cannot be excluded that ambroxol could stimulate lysosomal-dependent pathway of protein clearance in ALS (Boland et al., 2018), as ambroxol reduces the alpha-synucleinopathy in an *in vivo* model of PD (Migdalska‐Richards et al., 2016b).

Gaucher’s disease is caused by genetic mutations of GBA1, resulting in the loss of GBA1 activity (Kanfer et al., 1975; Vardi et al., 2016). In addition, mutations on GBA1 and/or reduced expression of the protein are an important risk factor of PD (Barrett et al., 2013). 

Recently, high dose of ambroxol was able to decrease the severity of neurological symptoms of patients with the neuropathic form of Gaucher’s disease, suggesting that oral administration of ambroxol successfully target the metabolism of glycosphingolipids in the central nervous system (Narita et al., 2016). Two other clinical trials are currently investigating the effects of ambroxol in PD (NCT02941822; NCT02914366). 

### Ambroxol Improved the Plasticity of Motor Units

We have also shown that ambroxol hastened functional recovery in a non-transgenic animal model of sciatic nerve injury. Given the modest effect on the kinetic of recovery, the effects of ambroxol are most likely due to an increased ability to rebuilt NMJs. Indeed, in presence of ambroxol, the percentage of re-innervated NMJs was twice higher than that in the vehicle group. Moreover, the *in vitro* co-culture model of motor units provided similar results as ambroxol strongly increased the formation of NMJs but modestly promoted axonal elongation. It indicates that ambroxol was able to stimulate the formation of NMJs. This effect could explain the beneficial outcomes in the SOD1^G86R^ mice through motor axonal sprouting and muscle re-innervation. 

Altogether, our results further connected the glycosphingolipid metabolism to the pathophysiology of ALS and indicated that inhibition of GBA2 may be a novel target for ALS.

## Methods

### Animal Care and Maintenance

FVB/N non-transgenic female mice derived from Charles River were used to perform nerve sciatic injury experiment. 

For others’ experiments, FVB/N female mice, overexpressing the SOD1^G86R^ (Ripps et al., 1995), were generated and maintained in our animal facility at 23°C with 12-h light/dark cycle. They had water and regular A04 rodent chow *ad libitum*. AMB (3 mM, Sigma-Aldrich) was given by drinking water for mice treated, and vehicle mice was treated with animal facility water. For the presymptomatic stage study, the treatment started at 75 days of age and stopped at 95 days of age. For the symptomatic stage study (survival experiment), the treatment started to 95 days of age and stopped when mice were euthanized. Mice showing strong motor dysfunction at 93 days of age were not included in the symptomatic cohort. Mice were euthanized when animals were paralyzed and unable to roll over within 5 s after being pushed on their back. For euthanasia, mice were intracardially perfused with PBS 1X at 4°C after intraperitoneal injection with Dolethal (120 mg/kg).

### Motor Assessment 

Body mass was analyzed on a daily basis. Muscle strength (mean of three tests, grip test, Bioseb, Chaville, France) and the inverted grid test to assess the motor performance and coordination of mice were analyzed every 2 days. Onset of muscle strength loss was defined as a drop of more than 10% of the mouse maximal strength. 

### Sciatic Nerve Injury

Peripheral nerve injury was performed in order to induce muscle denervation and axonal regeneration. Wild-type mice were anesthetized with ketamine chlorohydrate (100 mg/kg) and xylazine (5 mg/kg). The sciatic nerve was exposed at mid-thigh level and lesioned with fine forceps for 30 s. The skin incision was sutured, and mice were allowed to recover. The hind limb, contralateral to the lesion, served as control. Mice were treated with AMB (3 mM) for 13 days, starting the day before surgery. Mice were followed on a daily basis. Mice were sacrificed by intraperitoneal injection with Doléthal (120 mg/kg) and intracardially perfused with PBS at 4°C.

### Retrograde Labeling

Mice were treated with AMB (3 mM in drinking water) or with the vehicle solution (regular drinking water) from 75d of age at 95d of age. Mice were anesthetized at 94 days of age with ketamine chlorohydrate (100 mg/kg) and xylazine (5 mg/kg), and their hind limb muscles were injected with fluorogold (hydroxystilbamidine bis[methanesulfonate], Sigma-Aldrich; 10 mg/ml in PBS, 10% DMSO) FG with Hamilton seringue (26 gauge, 10 µl), and tissues were collected 24 h after injection. 

### Motor Neurons Counting

Tissues were fixed paraformaldehyde 4% and stored in PBS at 4°C until further use. Lumbar segments L1–L3 fixed in paraformaldehyde 4% were used for studying the number of motor neurons innervating hind limb muscle. After cryoprotection in 30% sucrose, coronal sections 16 μm thick from L1–L3 spinal segment were realized with a cryostat and were either stained with an anti-choline acetylcholine transferase (1/100, Millipore, France) and an alexa594-conjugated goat (1/200, Jackson ImmunoResearch, Suffolk, UK) antibodies, either mount on slides for Fluorogold counting. All neurons located in the ventral horn, which were >400 μm² in size and ChAT-positive, were considered as alpha motor neurons. 10 sections of spinal cord were counted, and cell area of motor neurons was measured with ApoTome 2 (Zeiss) microscope. 

### Neuromuscular Junction Labeling

Tissues were fixed paraformaldehyde 4% and stored in PBS at 4°C until further use. Under a binocular microscope, tibialis anterior muscle fibers were prepared into thin bundles. Neuromuscular junction morphology was studied by labeling of the acetylcholine receptors with rhodamine-conjugated α-bungarotoxin (1/400, Sigma-Aldrich), and labeling of nerve terminals was performed with a rabbit polyclonal anti-synaptophysin antibody diluted 1/50 (Abcam, Cambridge, UK), and anti-neurofilament diluted 1/50. For immunofluorescence of terminal nerve, Alexa-conjugated goat anti-rabbit IgG diluted 1/500 (Jackson ImmunoResearch, Suffolk, UK) was used. Muscle bundles were mounted into slides, prior to fluorescence microscopy (ApoTome 2, Zeiss). NMJs were considered as denervated when the presynaptic nerve terminal was absent from the postsynaptic region. 

### Beta-Glucosidase Activity

Tissues were snap frozen in liquid nitrogen and stored at −80°C until further use. Frozen tissues were lysed with a TissuLyser (Qiagen, CA) and suspended in phosphate potassium (pH7) buffer extraction. After centrifugation (12,000 rpm for 15 min at 4°C), the supernatants were transferred to new tubes and stored at −80°C. The bicinchoninic acid assay (BCA) method, a colorimetric assay method, was used to measured protein level with a BCA range (Interchim). A GBA buffer was used and adapted according to the enzymatic activity desired (McIlvaine buffer, 150 mM). For GBA1, enzymatic reaction was carried out with the GBA buffer pH4.6 and adding 0.1% triton and 0.1% BSA. For GBA2, enzymatic reaction was carried out with the GBA buffer pH5.8 and adding 0.1% BSA. After inhibitor addition and fluorescent agent 4-methylumbelliferone addition (4-MU, Sigma-Aldrich), samples were incubated 2 h at 37°C. For reading results, excitation was carried out at 360-nm excitation, and absorbance was measured at 445 nm emission (TriStar LB 941, Berthold Technologies).

### Co-Culture of Spinal Cord Explants and Myoblasts

The rat spinal cord–human muscle co-culture was performed as described previously (Combes et al., 2015). Human myoblast was grown on 96-well plate in a mix of MEM and M199 medium, supplemented with glutamine 2 mM, insulin, epidermal growth factor, basic fibroblast growth factor, fetal bovine serum 10%, and penicillin/streptomycin. Spinal cord explants with dorsal roots of 13-day-old Wistar rat embryos (Janvier, Le Genest-St-IsIe, France) were dissected and grown on the muscle monolayer. The co-cultures were maintained in a mix medium (MEM/M199), with 5% fetal calf serum, insulin, glutamine, and penicillin streptomycin. Ambroxol treatment was initiated when the spinal cord explants were added to the muscle monolayer and lasted 27 days. The treatment was renewed every other day during medium change. A total of 10 wells per conditions have been initiated. *In vitro* NMJs and neurite network were identified by immunostaining with alpha-bungarotoxin coupled with Alexa 488 and with a primary antibody against neurofilament 200 kD from mouse revealed with an anti-mouse Alexa 568. Sixty pictures have been automatically taken using same acquisition parameter with ImageXpress (Molecular Device) at 10× magnification and were analyzed with MetaXpress (Molecular Device). After segmentation of the pictures (**Supplementary Figure 3**), the total length of the neurite network and the total area of clustered nAChR were measured.

### Statistical Analysis

Data are expressed as the mean ± SEM and were analyzed with GraphPad Prism version 6.0 software. Student’s *t* test was used to compare two groups, and ANOVA followed by Fisher’s LSD test was applied to compare more than two groups. Grip strength curves were analyzed with two-way ANOVA, and survival curves were analyzed with log-rank test. Differences with *p*-values of <0.05 were considered significant.

## Data Availability

The raw data supporting the conclusions of this manuscript will be made available by the authors, without undue reservation, to any qualified researcher.

## Ethics Statement

Experiments were performed by authorized investigator after approval by the ethic committee of the University of Strasbourg and by the ministry of higher education and research (APAFIS #4555; #7146; #8828; #17157). They followed current European Union regulations (Directive 2010/63/EU).

## Author Contributions

Conceived and designed the experiments: AB, J-PL, AH; Performed the experiments: AB, CQ, AM, AH; Analysis and interpretation of the data: AB, NC, MS, J-PL, AH; Contributed reagents/materials/analysis tools: MS, J-PL; Wrote, discussed and approved the final version of the manuscript: AB, CQ, AM, NC, MS, J-PL, AH.

## Funding

The work was funded by “Association pour la Recherche et le Développement de Moyens de Lutte contre les Maladies Neurodégénératives” (AREMANE, J.P.L.), the “Association française contre les myopathies” (AFM, JPL). SRS contributed with salary and reagents. J-PL and MS are currently receiving funds from Fight MND foundation.

## Conflict of Interest Statement

MS, J-PL, and AH hold a patent entitled ‘Inhibitors of GlcCer degradation in the treatment of diseases of the motor units’, and the European Medicines Agency has granted AMB orphan designation for the therapy of amyotrophic lateral sclerosis to Spedding Research Solutions SAS. AH worked for part of the period of this work for Spedding Research Solutions SAS, where MS is president. AH and NC are currently employed by Neuro-sys SAS.

The remaining authors declare that the research was conducted in the absence of any commercial or financial relationships that could be construed as a potential conflict of interest.
