# Supplementary material for: Ambroxol Hydrochloride Improves Motor Functions and Extends Survival in a Mouse Model of Familial Amyotrophic Lateral Sclerosis
Source: Front Pharmacol. 2019 Aug 7;10:883. doi: 10.3389/fphar.2019.00883 (PMC6692493; doi:10.3389/fphar.2019.00883)
Supplement: Supplementary file 1 [file DataSheet_1.pdf]

Supplementary figure

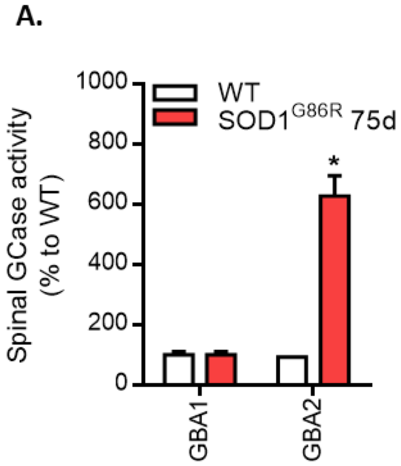

Supplementary figure 1. Tissue GBA1 and GBA2 activities in presymptomatic SOD1^{G86R} mice. (A) Basal GCase activity in spinal cord SOD1^{G86R} and WT mice at 75d (n=3/group, *p<0,05).

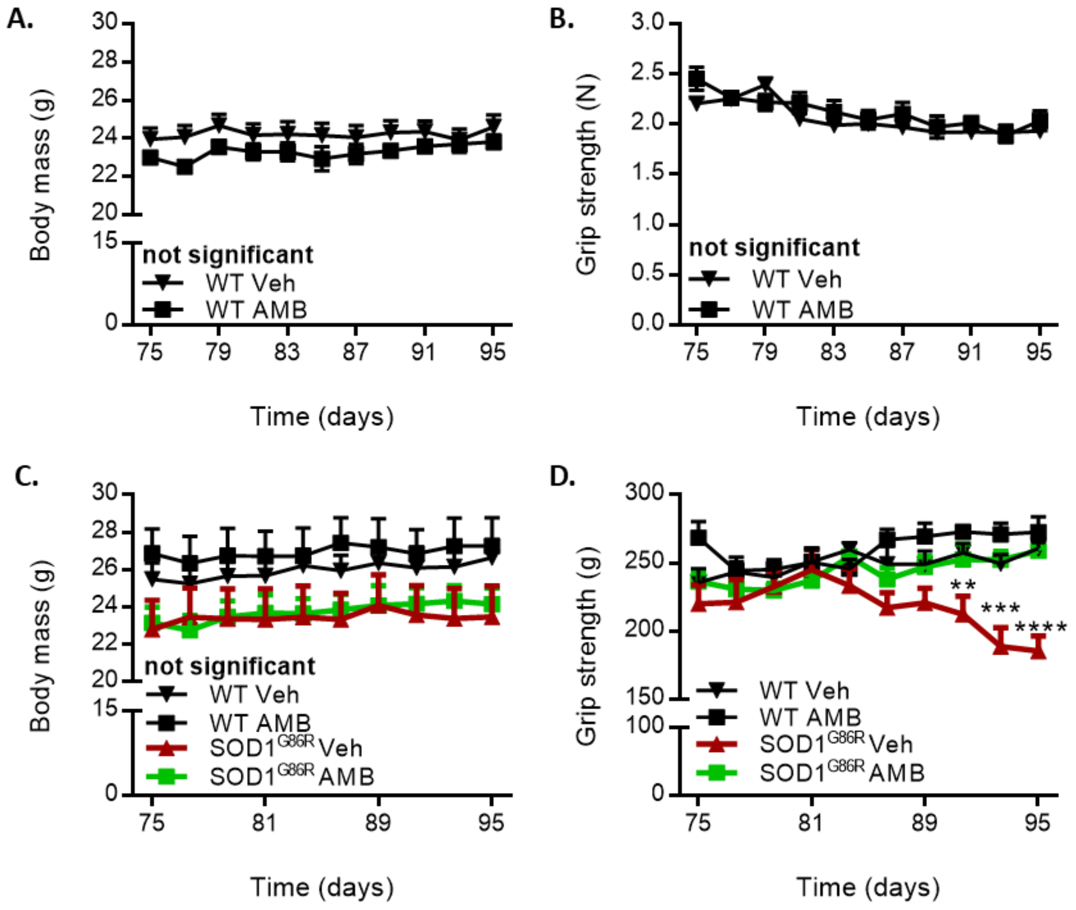

Supplementary figure 2. AMB improves motor functions and delays disease onset in pre-symptomatic SOD1^{G86R} mice. (A) Body mass evolution in WT mice after AMB treatment (not significant, n=7-8/group). **(B)** Muscle strength evolution in WT mice after AMB treatment (not significant, n=7-8/group). **(C)** Body mass evolution in other cohort of SOD1^{G86R} mice and WT mice (not significant, n=5/group). **(D)** Muscle strength evolution in other cohort of SOD1^{G86R} mice and WT mice (SOD1^{G86R} Veh vs AMB, **p<0,01; ***p<0,001, n=5/group)

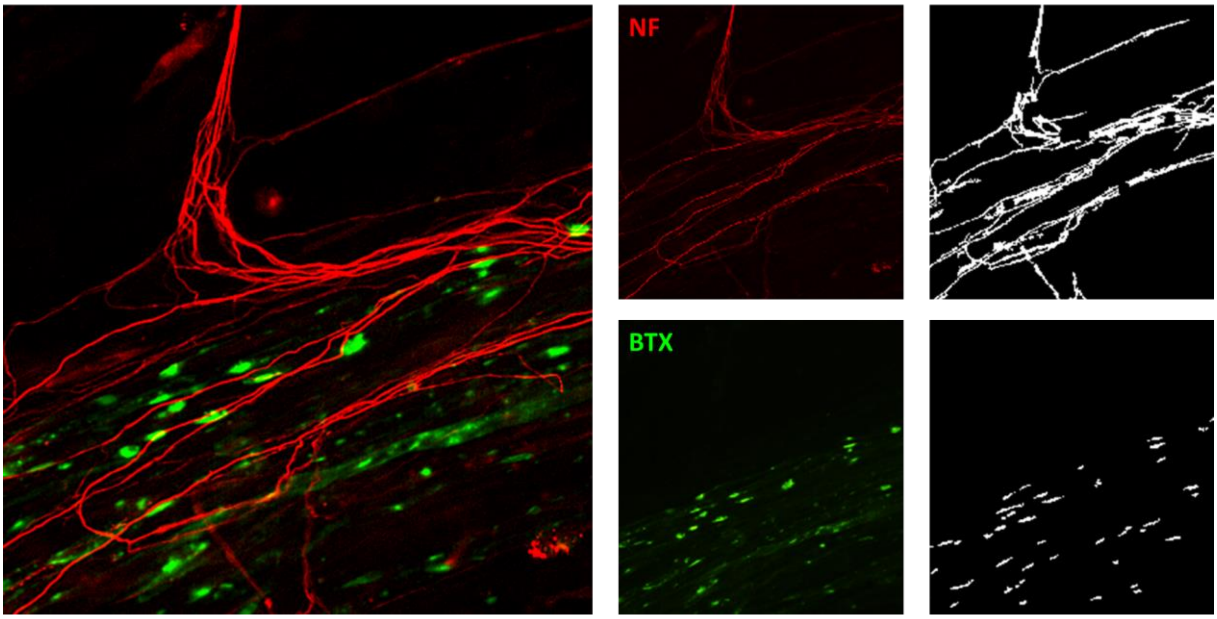

Supplementary figure 3. Computer-assisted analysis of neurite network and NMJs, visualized by the clusterization of nAChRs.

Example of signal segmentation from a picture of a co-culture of spinal explant and human myoblasts (left). Neurofilament is shown in red and nAChRs clusters are shown in green. The segmentation of the neurite network (upper right panel) or the segmentation of the NMJs (lower right panel) is automatically performed by the software (MetaXpress, Molecular Device).

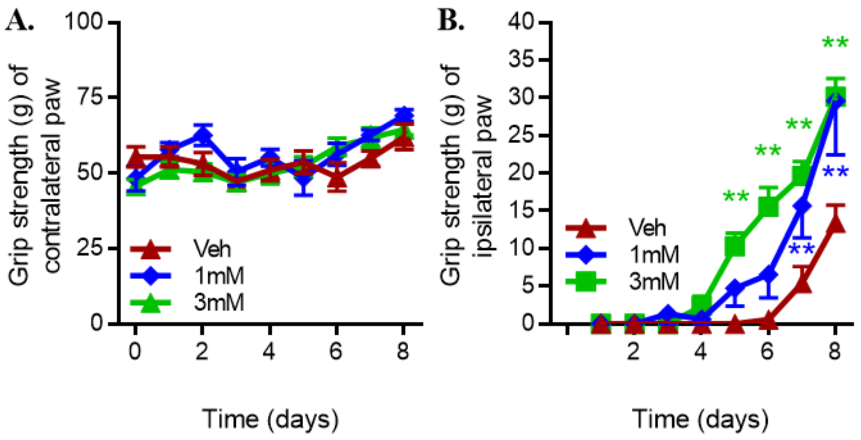

Supplementary figure 4. AMB improves in vivo axonal plasticity and nerve regeneration after sciatic nerve crush study in an other non-transgenic mice. (A) Muscle strength of contralateral hind paws (n=7-10/group, not significant). **(B)** Muscle strength of ipsilateral hind paws (n=7-10/group, **p<0,0001, green stars: AMB 3mM versus vehicle; blue stars: AMB 1mM versus vehicle).
